# Demand Response Contextual Remuneration of Prosumers with Distributed Storage

**DOI:** 10.3390/s22228877

**Published:** 2022-11-17

**Authors:** Cátia Silva, Pedro Faria, Bruno Ribeiro, Luís Gomes, Zita Vale

**Affiliations:** Research Group on Intelligent Engineering and Computing for Advanced Innovation and Development (GECAD), Intelligent Systems Associated Laboratory (LASI), Polytechnic of Porto, 4200-072 Porto, Portugal

**Keywords:** contextual remuneration, battery energy storage, demand response, optimal management, smart grids

## Abstract

Prosumers are emerging in the power and energy market to provide load flexibility to smooth the use of distributed generation. The volatile behavior increases the production prediction complexity, and the demand side must take a step forward to participate in demand response events triggered by a community manager. If balance is achieved, the participants should be compensated for the discomfort caused. The authors in this paper propose a methodology to optimally manage a community, with a focus on the remuneration of community members for the provided flexibility. Four approaches were compared and evaluated, considering contextual tariffs. The obtained results show that it was possible to improve the fairness of the remuneration, which is an incentive and compensation for the loss of comfort. The single fair remuneration approach was more beneficial to the community manager, since the total remuneration was lower than the remaining approaches (163.81 m.u. in case study 3). From the prosumers’ side, considering a clustering method was more advantageous, since higher remuneration was distributed for the flexibility provided (196.27 m.u. in case study 3).

## 1. Introduction

### 1.1. Background

Climate change demands a change in the power and energy sector. It is essential to find solutions that properly introduce these greener approaches to reduce fossil fuel use and decrease air pollution and greenhouse effects [1]. The smart grid concept focuses on the consumer side and their flexibility. However, management tools and knowledge must be provided to take a step forward towards successful implementation in the real market [2].

### 1.2. Challenges

Although slow, there have been advances in this area [3]. However, the management of energy systems is more complex due to the integration of Distributed Generation (DG), namely renewable-based resources with their volatile behavior, and the addition of energy prosumers as players in energy market transactions [4]. Not only do they provide flexibility by participating in Demand Response (DR) events with their appliances, but also as prosumers, with DG to suppress their own needs or sell their surplus [5]. Still, the flexibility provided by these new and active communities should be properly managed to avoid consumer discomfort, and reduce response uncertainty, rebound effect, and unsupplied power, among other grid problems [6]. It becomes crucial to define a tool to optimally manage resource scheduling to surpass this challenge; namely, using the BES capacity.

Many solutions are being developed to deal with the complex management of local communities and their new and complex resources [7]. Indeed, with the concept of the Internet of Things (IoT), different equipment (sensors and actuators, among others) can be applied to gather information on the consumer side and provide proper solutions [8]. Nevertheless, dealing with big data is one of the major issues in the literature, but progress has been made [9,10]. Prabadevi B et al. [11] analyze the possibility of deep learning approaches for tackling this challenge, leaving the inaccuracies in electricity load forecast as an open issue, where the lack of evaluation of real-time data was at issue. According to these authors, information (both historical and real-time data) is crucial for making wise and optimal decisions, so there is a challenge for the community manager: decide which knowledge is useful since, as mentioned, real-time approaches can be challenging and require fast responses to grid issues.

### 1.3. Literature Review

System operators require a scheduling model that considers security and economic issues when using energy storage systems, as Meysam Khojasteh et al. [12] highlight. These authors proposed a linear optimization model for synchronous generators and BES in the collective energy and reserve market. Their proposal reduced the total operating costs and provided adequate security by deploying energy storage systems. In [13], the authors introduced the aggregated scheduling of energy storage systems and wind power resources in the same joint markets. For this case study, results indicate that the day-ahead and real-time markets can be considered the optimal options for buying and selling energy storage systems’ energy.

Nevertheless, sharing a large capacity battery across a group of small prosumers in a community can alleviate the economic deterrents but also exploit the fact that behavior patterns do not necessarily overlap, as Jiyun Yao and Parv Venkitasubramaniam [14] believe. However, this introduces competition [15]. These authors introduce a stochastic, general-sum, game-theoretical framework to solve this problem and capture the competitive behaviors managing charging and discharging based on the received, sometimes incomplete, information. The results provided a close-to-optimal performance using the strategy with real electricity usage and pricing data.

With this, it is crucial to take a step forward to implement energy storage systems in local communities, namely the ones with residential prosumers with renewable-based resources, as a means to increase their self-consumption [16]. Lisa Calearo et al. [17] stress this fact in their work, where a comparison was made between the benefits of a PV prosumer with an EV under two options: installing a BES or applying smart charging. Furthermore, Farzad Arasteh and Gholam H. Riahy [18] developed an online model-based predictive-control approach for optimal real-time operation of wind-integrated power systems, including DR and energy storage systems facilities. In the results, the authors could reduce operational costs through optimal uncertainty management.

The proper knowledge must be selected from a large amount of data provided by IoT devices. In the study from [19], the authors used a contextual approach to improve the accuracy of aggregated schedules considering DR performances from previous event experiences in the same context (weather conditions and period). The goal was to understand which factor impacts the final performance rate attributed to participants and improve the overall method. However, only direct load control and load appliances were contemplated in the DR events, disregarding prosumers. A previous work [20] did contemplate this type of prosumer. Still, the main goal was fair remuneration for DR participants by understanding the benefits of considering them as individuals or unique players using clustering methods. Along the same line, the study developed [21] a remuneration structure comparing hierarchical and fuzzy c-means, considering the maximum tariff in each group to compensate the DR participants. However, the participant trustworthiness level was not considered; the prosumers have uncertain behaviors and might not participate as requested. Considering them as economic and rational agents might lead to misleading results [22].

Table 1 summarizes the important topics referred to in the literature reviewed previously, considered to be used for motivation for the current work. Regarding the table: prosumers were rarely used; the main Demand Response programs used are incentive-based; the chosen energy storage system is the battery; the horizon most used in these works is real-time; only one work considers fair remuneration of DR participants.

In the present paper, the authors aim to gather all these concepts with the proposed methodology.

### 1.4. Scopes

The present study falls into the scope of optimal resource scheduling, considering a contextual approach for DR event participants. In this way, the authors aim to address and solve problems related to fair and contextual remuneration, reduce response uncertainty, BES optimal management, and self-consumption.

### 1.5. Motivation

Considering the works analyzed previously, the authors’ main motivation is to provide innovative contributions for local community management of several resources with uncertain and volatile behavior. Furthermore, dealing with big data issues by selecting contextual information could be useful in characterizing DR performances. And finally, the authors contemplate not only load flexibility prosumers, but also prosumers with more resources beneficial for the grid functioning, properly managed and well-motivated by using fair and contextual remuneration.

### 1.6. Contributions

With this motivation and the mentioned challenges, the authors address the problem in this paper: how to fairly remunerate prosumers for participating in DR events providing flexibility with several resources and considering different energy price schemes. The following features are listed as innovative aspects of the methodology proposed in the present paper:

- Optimizing prosumer behavior in a distributed management, considering past DR events to predict and classify their response to an event;
- Evaluate several approaches to increase remuneration fairness;
- Compare several energy price schemes to understand the benefits from the community manager and the prosumer perspectives.

### 1.7. Organization of Paper

The present paper is organized into five main sections. Section 1 presents the present work’s background, motivations, related works, and innovations. Then in Section 2, the proposed methodology is presented in detail. The case studies and several scenarios are presented in Section 3 to be further discussed in the results and discussion section. Finally, Section 5 brings forth the conclusions from this study.

## 2. Proposed Methodology

**Figure 1:**
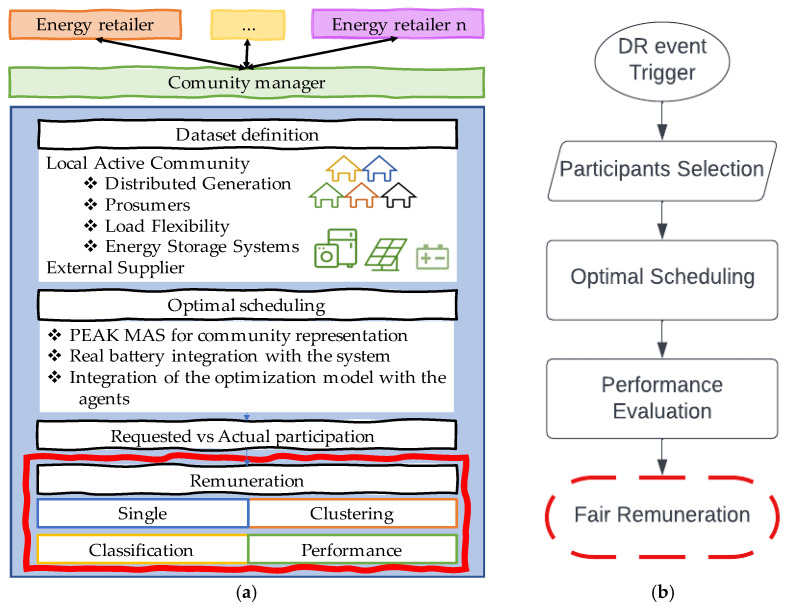
Proposed methodology: (**a**) diagram, (**b**) flowchart.

The authors developed a methodology to optimally manage the resources in a community, such as Distributed Generation (DG), prosumers, load flexibility, and energy storage systems. Believing that context is an important matter, bringing intelligence to the models developed, the authors believe introducing this concept will improve the solution compared with previous works [23]. For the present paper, besides the inclusion of BES in the prosumer’s portfolio, the authors intend to evaluate ways to motivate participation in DR events and reduce the operating costs from the community manager by selecting only the proper participants for a certain context, following the proposed methodology in Figure 1.

For a community manager, several offers are being made from the energy retailers as a competitive market is introduced. Furthermore, dealing with the new and uninformed prosumers as market players with a more direct impact on the market transactions increases the complexity of managing active communities.

**Figure 2:**
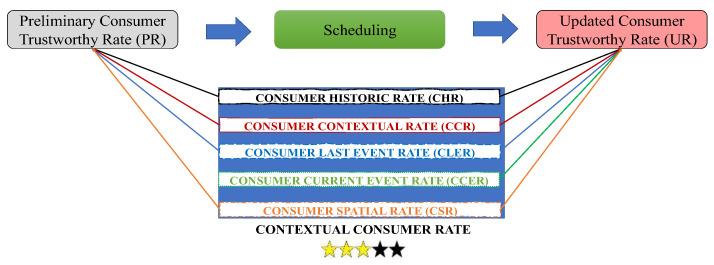
CTR in the scope of the proposed methodology.

Following Figure 1b, the participants are selected to attend, starting with the DR event triggered in a specific context. To maintain continuity with previous works, the authors used a rate designed to classify the participants’ performance on a DR event: Consumer Trustworthy Rate (CTR), defined according to Figure 2. Three yellow stars in Figure 2 means three out of five.

Before scheduling, information is gathered regarding DR events in the same context—for instance, the same hour, day of the week, or even the temperature registered for each participant. This rate will be useful for understanding which prosumers are more willing to participate in each situation. However, privacy matters could be raised since prosumers might share sensitive information regarding their appliances to participate in DR events. Before the event, the community manager and the participants should agree on the data provided, since this is a contextual approach. For instance, in load-shifting programs, the appliance schedule might be shared to be optimally attributed to a different period, avoiding consumer discomfort and rebound effect.

To avoid privacy issues, the proposed methodology-optimal schedule in the present paper is performed in a distributed way, so the aggregator does not have any private information on a real-time basis or any other horizon. Each prosumer building does their own internal management with the optimal schedule proposed and does not provide private information regarding a specific appliance’s availability or behavior. The aggregator has only the sum of actual flexibility response from each consumer at the end of the month to update the CTR and proceed for DR participation remuneration.

Participants with high values of CTR are usually the more trustworthy. The formulation of the CTR changes according to the needed step: before (preliminary consumer trustworthy rate as PR) or after (updated consumer trustworthy rate as UR) the scheduling. To select the participants for DR events, the PR is defined according to the consumer historic rate (CHR), the consumer contextual rate (CCR), the consumer last event rate (CLER), and the consumer spatial rate (CSR). The first one is the average of the last five performances in the same period. CCR is divided into two different perspectives: time and weather. The participant’s performance according to these indicators will result in the CCR. After that, the CLER differs from the CHR to update the CTR according to the last performance. Finally, CSR is only used if the aggregator has knowledge regarding the participant’s current location on the grid. For cases where a voltage violation is detected, the participants closer to the faulty bus must have priority. In the present paper, CSR was not considered since it was not the focus of the study. Further privacy issues regarding CTR were already discussed by the authors in prior works with additional consumer information [24], so this topic will not be approached in the present paper. After the scheduling is performed and the comparison between the requested and the actual flexibility is made, the CTR must be updated with the performance from the current period—CCER.

As soon as the participants are selected, the optimal scheduling phase starts. Equation (1) represents the objective function from the mixed-integer linear programming optimization. Since the tariffs are defined hourly, the term ∆t was added to adjust the consumption for a different time basis.

(1) $$\min EB=\overset{T}{\underset{t-1}{\sum }}[\left(P_{\left(t\right)}^{grid_{in}}.C_{\left(t\right)}^{grid_{in}}-P_{\left(t\right)}^{grid_{out}}.C_{\left(t\right)}^{grid_{out}}\right).\frac{1}{\Delta t}+\overset{C}{\underset{c=1}{\sum }}P_{\left(c,t\right)}^{DR}.W_{\left(c,t\right)}^{DR}]\;\{\begin{array}{c} P_{\left(t\right)}^{grid_{in}}=P_{\left(t\right)}^{grid},\;if\;P_{\left(t\right)}^{grid}>0\\ P_{\left(t\right)}^{grid_{out}}=P_{\left(t\right)}^{grid},\;if\;P_{\left(t\right)}^{grid}<0\\ \forall t\in \left\{1,\ldots ,T\right\} \end{array}$$

Equation (2) represents the balance that must exist between the consumption and generation resources in the community. Equation (3) shows the upper and lower limits for grid usage. As can be seen, the sign of $P_{\left(t\right)}^{grid}$ changes according to the transactions done: if the energy is bought, the value is positive. Otherwise, it is negative.

(2) $$\overset{P}{\underset{p=1}{\sum }}P_{\left(p,t\right)}^{PV}+P_{\left(t\right)}^{grid}+\overset{C}{\underset{c=1}{\sum }}P_{\left(c,t\right)}^{DR}+\overset{S}{\underset{s=1}{\sum }}P_{\left(s,t\right)}^{dch}=P_{\left(t\right)}^{load}+\overset{S}{\underset{s=1}{\sum }}P_{\left(s,t\right)}^{ch},\forall t\in \left\{1,\ldots ,T\right\}$$

(3) $$-P_{\left(t\right)}^{gridmax_{out}}\leq P_{\left(t\right)}^{grid}\leq P_{\left(t\right)}^{gridmax_{in}},\forall t\in \left\{1,\ldots ,T\right\}$$

Regarding the flexibility provided by the participants in the DR events, it is represented with $P_{\left(c,t\right)}^{DR}$ and is limited according to upper and lower limits shown on Equation (4). Also, the authors considered that loads are connected to relays and only when activated—using the binary variable $X_{\left(c,t\right)}^{DR}$, the loads can be shed according to Equation (5).

(4) $$0\leq P_{\left(c,t\right)}^{DR}\leq P_{\left(c,t\right)}^{DRmax},\forall t\in \left\{1,\ldots ,T\right\},c\in \left\{1,\ldots ,C\right\}$$

(5) $$P_{\left(c,t\right)}^{DR}=P_{\left(c,t\right)}^{DRmax}.X_{\left(c,t\right)}^{DR},X_{\left(c,t\right)}^{DR}\in \left\{0,1\right\},\forall t\in \left\{1,\ldots ,T\right\},c\in \left\{1,\ldots ,C\right\}$$

Energy storage systems considered in the methodology are limited by several constraints represented from Equations (6)–(10). Equation (6) represents the upper and lower limits of the energy storage system operation capacity. Equations (7) and (8) represent the charge and discharge limits per period, respectively, associated with binary variables. So with Equation (9), the aggregator can guarantee the impossibility of charging and discharging with these binary variables $X_{\left(s,t\right)}^{ch}$ and $X_{\left(s,t\right)}^{dch}\;$in the same period. Finally, Equation (10) represents the state of charge of the energy storage system maintaining the power balance—the sum of the previous state and what was charged or what was discharged for the current period.

(6) $$E_{\left(s,t\right)}^{stormin}\leq E_{\left(s,t\right)}^{stor}\leq E_{\left(s,t\right)}^{stormax},\forall t\in \left\{1,\ldots ,T\right\},s\in \left\{1,\ldots ,S\right\}$$

(7) $$0\leq P_{\left(s,t\right)}^{ch}\leq P_{\left(s,t\right)}^{chmax}.X_{\left(s,t\right)}^{ch},X_{\left(s,t\right)}^{ch}\in \left\{0,1\right\},\forall t\in \left\{1,\ldots ,T\right\},s\in \left\{1,\ldots ,S\right\}$$

(8) $$0\leq P_{\left(s,t\right)}^{dch}\leq P_{\left(s,t\right)}^{dchmax}.X_{\left(s,t\right)}^{dch},X_{\left(s,t\right)}^{dch}\in \left\{0,1\right\},\forall t\in \left\{1,\ldots ,T\right\},s\in \left\{1,\ldots ,S\right\}$$

(9) $$X_{\left(s,t\right)}^{dch}+X_{\left(s,t\right)}^{ch}\leq 1,\forall t\in \left\{1,\ldots ,T\right\},s\in \left\{1,\ldots ,S\right\}$$

(10) $$E_{\left(s,t\right)}^{stor}=E_{\left(s,t-1\right)}^{stor}+P_{\left(s,t\right)}^{ch}+P_{\left(s,t\right)}^{dch},\forall t\in \left\{1,\ldots ,T\right\},s\in \left\{1,\ldots ,S\right\}$$

Once finished with the scheduling, the following stage is represented on Figure 2b as “Performance Evaluation”, where a comparison between the actual and the requested participation is made to properly assign the remuneration tariffs to the ones that contributed to the DR event.

The fair remuneration phase is the focus of this study. The authors proposed four approaches: single, clustering, classification, and performance. The single approach considers that flexibility is remunerated according to the energy price applied for the current period. Several schedules were considered for dividing, for instance, the day into peak, valley, and off-valley periods. Each period has a different tariff, and the flexibility is compensated with the same value.

For the clustering approach, the authors opt for one of the well-known partitional methods—k-means. The algorithm aims to find a centroid value representing each group, comparing the distance between elements until the minimum value is found. The k-means clustering method was already studied and widely used with various extensions in the literature. For the proposed methodology, the authors intend to aggregate prosumers with similar flexibility profiles and remunerate higher values with better tariffs, which may lead to different tariff values per period, since the group with higher flexibility can also change. By getting the output data from the method in which a group is assigned to each participant, the authors want to create the proper rules and then attribute the group to other participants without performing the clustering method each period. In this way, classification methods can be used. Three different methods were tested within the scope of the present paper: decision tree, k-nearest neighbors (KNN), and artificial neural networks (ANN). These methods were then compared and evaluated using accuracy and mean absolute error (MAE).

Finally, the UR is used for the remuneration since it contains the information from the actual performance. Each rate is tariff-associated, and the higher the rate, the better the compensation.

The novelty from previous works relies on the contextual change of energy price and remuneration. The authors believe proper compensation is crucial to motivate continuous participation and make this transaction known to prosumers. It will take time, education, and resources to make the prosumers better power and energy market players. Still, the authors believe continuous participation and experience will also be important to progress. With higher knowledge regarding the community come better results in managing community resources. It will also benefit the prosumers, since their load can be changed to other periods when the energy price is lower. Comparing several approaches to remunerate properly and fairly can be useful for developing a tool capable of taking a step forward to apply DR in the real market with optimal results.

## 3. Case Study

To test and validate the optimization model, it was used with a novel multi-agent system conceived and developed by the authors called Python-based Ecosystem for Agent Communities (PEAK) [25]. PEAK is a multi-agent framework that aims to support and manage the development of multi-agent ecosystems in a simple way, based on the Python programming language. This framework can create simulation environments and provide a feasible solution for a pilot deployment. PEAK can integrate real devices, such as energy resources, loads, and IoT devices, using specific drivers for each communication protocol. Furthermore, it is possible to integrate mathematical and machine learning models within the agents. By default, a system created with PEAK registers every interaction inside the ecosystem for posterior debugging and analysis.

The paper uses Multi-Agent Systems (MAS) to model and represent smart grid entities. The authors consider the approach a good fit, as they can decentralize the computational effort among agents. The proposed MAS solution uses the open-source PEAK framework (www.gecad.isep.ipp.pt/peak, accessed on 3 November 2022), which enables easy and fast development and deployment of MAS in simulated and real scenarios. As for the optimization model, it is distributed among agents, and each agent responsible for the optimization of its own resources.

**Figure 3:**
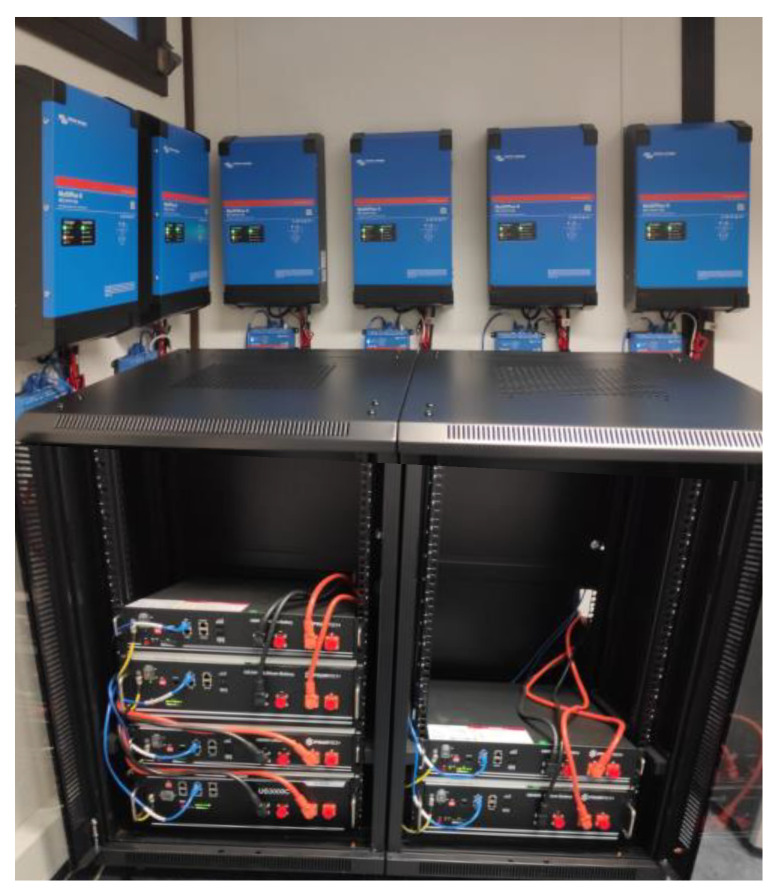
A real BESS from GECAD/ISEP was used for implementing the proposed methodology.

Energy storage in the literature has been identified with several options, such as ultra-capacitor, super magnetic, flywheel, compressed air, pumped hydro energy storage systems, and Battery Energy Storage Systems (BESS) [26]. In the present paper, BESS has been selected. As concluded in [26], combining BESS with DR can significantly reduce the size of conventional energy storage systems and improve power quality. For the present case study, a total of 19 prosumers/agents were considered, and five of those prosumers are connected to real BESS, while the others use simulated BESS, and both are constrained by (6) to (10). These real BESS belong to the GECAD/ISEP research center and can be seen in Figure 3, with a capacity of 2 kW. The developed PEAK agents for the proposed model do not represent a threat to the equipment when using data simulation. To avoid damage to the equipment, the PEAK was executed in real-time (running the case study for 24 h) while compliant with the physical limitations of BESS. Also, the BESS used, a Victron inverter, was configured to set charging, discharging, and state of the charge limits to avoid physical threats to the equipment. Regarding computational complexity, this framework uses multiprocessing and multithreading to increase simulation processing speed, enabling computation processing distribution between different machines.

At the beginning of the simulation, the real BESS starts at full capacity. Furthermore, to communicate with the BESS, the agents use the protocol ModBus/TCP. Regarding the optimization model, the authors integrated with the multi-agent system’s agents and optimized each period where the model is executed in each agent. The proposed model allows BESS configuration. In this way, for the case study in the present paper, the actual BESS may have a configuration like the simulated BESS.

**Figure 4:**
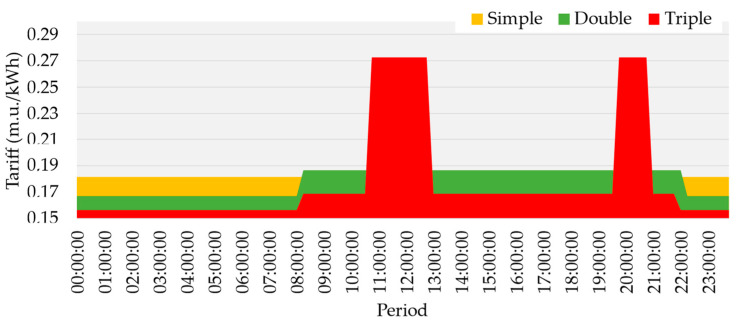
Schedule for the different tariffs.

Focusing on the contextual change of energy prices for resource scheduling, three case studies were created considering the tariff values from Table 2 and the schedule represented in Figure 4. The feed-in tariff was not considered for the case studies—values applied are currently considered by the main Portugal DSO. Only prosumers below 20.7 kW of contracted power were considered for this study. The 19 prosumers mentioned above with contracts as DR participants will provide flexibility. Moreover, these prosumers also use photovoltaic (PV) generation and a BESS associated with each one of them, being able to suppress their load consumption or sell the excess to the grid. The Portuguese DSO from which Table 2 tariffs were withdrawn considers two different schedule options: daily and weekly. However, this fact does not affect the tariff values, only the schedules in which they are applied; for the weekly schedule, the season matters.

The four remuneration approaches were then compared using the Figure 4 schedule for the different types of tariffs.

First, the single approach where the same tariffs from Table 2 are used for remunerating the participants in the DR event is the single approach where the same tariffs from Table 2 are used to remunerate the DR event participants. Then, a clustering approach is applied, aggregating the participants with similar behaviors. For this scenario, the number of clusters to be formed will equal three. The tariffs are attributed according to the context in which the DR event is triggered, following the schedule from Figure 4 according to the tri-hourly one. The classification scenario will create several rules to assign a tariff to the participants according to the context. And finally, the authors decided to apply a different approach. The CTR was used for the remuneration fees regarding participation in the DR event, as seen in Table 3.

Considering the values from Table 2, for the CTR scenario, the authors attribute the higher tariff value to each scheduled period (i.e., peak, off-valley, and valley) and divide according to the rate. So with higher rate values, the prosumer will receive more compensation for their participation, sometimes enough to pay for their load consumption for the current period and have profits. The approach’s main goal is to improve performance and encourage continuous participation, giving more benefits to the prosumer for participating in the market transactions. The dataset used for each case study is in Appendix A.

## 4. Results and Discussion

**Figure 5:**
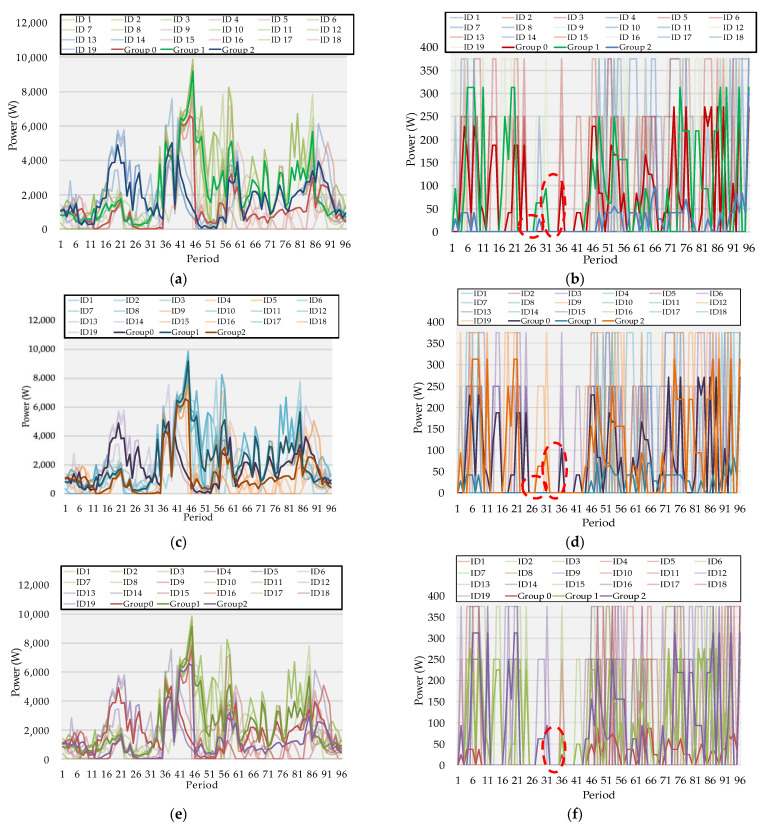
Scheduling results for case studies divided according to clustering method: (**a**) prosumers’ load flexibility per period for case study 1, (**b**) prosumers’ BES discharging per period for case study 1, (**c**) prosumers’ load flexibility per period for case study 2, (**d**) prosumers’ BES discharging per period for case study 2, (**e**) prosumers’ load flexibility per period for case study 3, (**f**) prosumers’ BES discharging per period for case study 3.

In the present section, the authors discuss the proposed methodology results when applied to the previously presented case study. The scheduling was performed for all the prosumers in the community considering (1) to (10). The prosumer resources flexible results can be seen in Figure 5a–f, from load reduction values (constrained by (4) and (5)) and BES discharging (constrained by (8)).

The results were then aggregated into three groups using the k-means clustering method to find the similarity between the load profiles. This consumer modeling technique was used by the authors for ease the analysis—the colors were also changed in the group according to each ID assigned, but with higher values of opacity.

Figure 5a,b represent the scheduling results for case study 1 (flat energy price tariff). Figure 5a clustering results show that Group 0 gathers the prosumers with low values of flexibility outside the peak zone, namely when compared with Group 2. From period 40 until period 58, Group 0 (represented by the red curve) had higher values of flexibility than Group 2 (represented by the blue curve), which prevailed mostly higher in the remaining periods. Regarding Group 1, before period 35, it was also below the blue line but remained superior to the others until around period 85. It must be highlighted that Group 0 had six prosumers, Group 1 gathered ten different participants, and the remaining were attributed to Group 2.

Results from Figure 5b achieved the maximum discharge value (375 W) for many periods and kept that value for no more than four consecutive periods. Nevertheless, the participant that contributed with this type of flexibility for the most periods was ID 16, keeping the maximum for four consecutive periods and then decreasing to 95 W. This participant was then assigned to Group 1, the four prosumers that have higher values of contribution for most of the time; namely, when there is no PV generation. Assigned to Group 0 were six different participants: ID2, ID3, ID5, ID6, ID13, and ID15. Group 2 has nine participants with lower values of contribution.

Figure 5c,d represent the scheduling results for the second case study that considers a bi-hourly tariff, meaning there are times of the day considered as valley and off-valley (including the peak hours) where the energy price is different, as seen in Table 2. As suggested by the Portuguese DSO, the bi-hourly tariff is ideal for the prosumers, with 40% of their daily consumption spent between 10 p.m. and 8 a.m. Still, the authors wanted to understand if these tariffs impact the flexibility in the community schedule—are two different energy tariffs enough to move the load consumption to lower price schedules?

Figure 5c flexibility results are similar to Figure 5a. Group 0 was assigned ID12, ID14, and ID19 (represented with purple curves), reducing by a total of 518 W for the whole day—the group with fewer members and less provided flexibility overall. For Group 1, the clustering method allocated ten participants to this group, which reduced the day by 2164 W. Finally, in Group 2, the reduction value was 773 W between the six participants within the group. Regarding the BES discharging results, some disparities can be found in Figure 5b. For instance, period 29 is highlighted with the red dotted circle on both figures—the discharging value from Group 2 in Figure 5b is null.

Figure 5e,f represent the results for the final case study where a tri-hourly tariff is considered, meaning three different values are assigned to periods, such as peak, off-valley, and valley, according to Table 2. However, applying the tri-hourly tariff did not affect the flexibility provided by the participants differently from the previous ones. Still, differences could be seen on the BES discharging chart marked with a red dotted circle in period 36, where the discharging value was slightly lower than the remaining cases.

**Figure 6:**
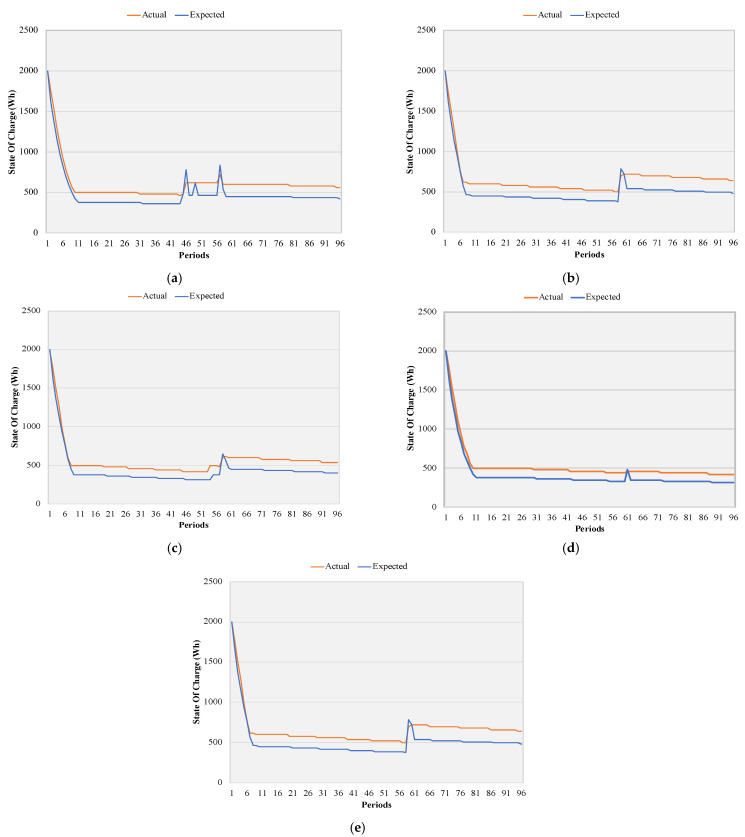
Comparison between the expected and the real state of charge from different batteries: (**a**) battery ID 0, (**b**) battery ID 1, (**c**) battery ID 2, (**d**) battery ID 3, (**e**) battery ID 4.

The simulated values had differences between the three case studies regarding the BESS perspective. To prove the viability and robustness of the proposed model, case study 3, with more tariffs, was tested in a real-time environment. The real state of charge for the five BES considered can be seen in Figure 6, according to the PEAK execution. When comparing both expected and real results, most of the time, the real values are above the expected, although it follows the same tendency. Regarding Figure 6a, between periods 40 and 60, a major difference can be seen between both curves—expected to charge and after discharge. Still, the real value kept above 500 W. Moving on to Figure 6b, from period 60, the battery was expected to discharge, but that did not happen in the real curve, similar to Figure 6c–e. The expected results were constrained by (7).

Moving on to the second phase of the study, regarding fair remuneration, the results for the first approach can be seen in Table 4 regarding the total flexibility of the prosumers’ load and the total BES discharging. By analyzing this table, the authors can conclude that the total compensation increased from case study 1 to case study 3. BES discharging remuneration decreased from case study 1 to case study 2 but achieved the maximum in case study 3 with 4.53 m.u.

When participating in DR events, the prosumers will benefit from changing their consumption to other periods with cheaper energy prices or even using PV resources for self-consumption. Furthermore, they will be fairly remunerated for helping with market transactions. For instance, according to Table 4, if the community prosumers did not participate, a maximum of 163.81 would be attributed, and grid problems might not be solved.

The results from the fair remuneration clustering approach can be seen in Figure 8 for both prosumers’ load flexibility and BESS discharging.

**Figure 7:**
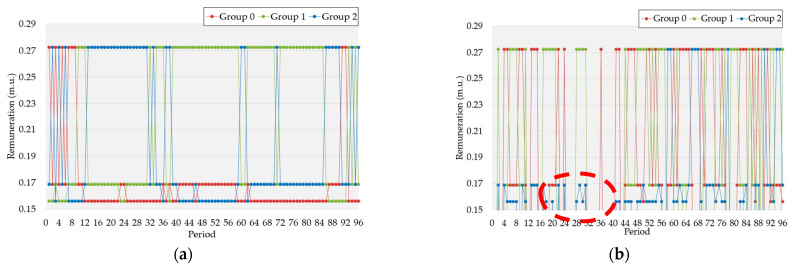

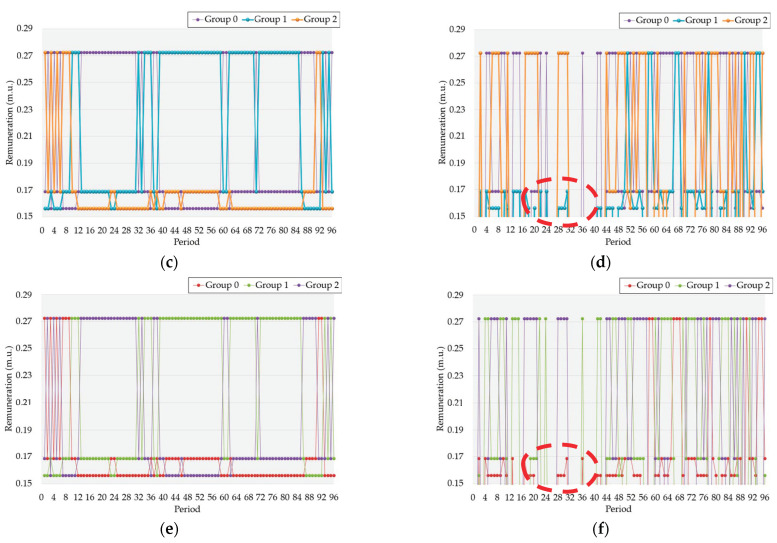
Contextual tariffs for case study 1 according to clustering results: (**a**) prosumers’ load flexibility remuneration per period for case study 1, (**b**) prosumers’ BES discharging remuneration per period for case study 1, (**c**) prosumers’ load flexibility remuneration per period for case study 2, (**d**) prosumers’ BES discharging remuneration per period for case study 2, (**e**) prosumers’ load flexibility remuneration per period for case study 3, (**f**) prosumers’ BES discharging remuneration per period for case study 3. The red circles highlight the same periods of Figure 5.

The remuneration tariffs were assigned according to groups with a higher value of flexibility for each period. In other words, the ones with higher accumulated flexibility will be assigned higher remuneration. It must be highlighted that remuneration tariffs were defined according to the tri-hourly tariff used previously. In this way, the group tariffs resulting from the prosumers’ flexibility are similar for the three case studies, according to Figure 7a,c,e. Disparities can be, however, found regarding the BES discharging results. Focusing again between periods 20 and 40, marked with a red dotted circle in Figure 7, there were differences between case studies. Firstly, for case study 1, group 2 changed tariffs twice—from 0.1561 (m.u./kW) to 0.1686 (m.u./kW). Regarding case study 2, group 1 increased their remuneration price from 0.1561 (m.u./kW) to 0.1686 (m.u./kW). Finally, similar to case study 2, group 0 from case study 3 increased the remuneration but also participated in period 36, achieving the second-best tariff. In contrast to the results from Figure 7, Table 5 was created to compare the daily remuneration obtained per group and case study.

As expected from the previous results, the remuneration from the actual flexibility of participants is the same for all the groups in the different case studies. However, the BES discharging results are different, demonstrating a higher gap between case study 3 and the remaining. Group 0 achieved a 2.75 m.u. for case study 1 and case study 2. Group 1 from case study 1 and Group 2 from case study 2 had similar remuneration of around 2.20 m.u. Group 2 from case study 3 received 2.22 m.u. The lowest remuneration was attributed to Group 2 from case study 1 (with 0.84 m.u.), Group 1 from case study 2 (with 0.87 m.u.), and Group 0 from case study 3 (with 1.08 m.u.). Regarding these results, from an overall perspective, opting for case study 3 can benefit the aggregator. These results indicate that adding more contextual information may reduce the total remuneration and still be fair with the participation compensation.

Furthermore, the authors used three different classification methods to create the proper tool to attribute the remuneration group to each participant, resorting to the results obtained from the clustering results. The classification method’s performance, using the selected indexes (accuracy and MAE), can be seen in Table 6. The results were obtained using Python libraries created for this goal. In examining the results and regarding the actual flexibility, the lowest accuracy value was achieved when the decision tree was performed—10.59%, with the 83.33% achieved with KNN. This method obtained better results from both accuracy and MAE for both datasets.

**Figure 8:**
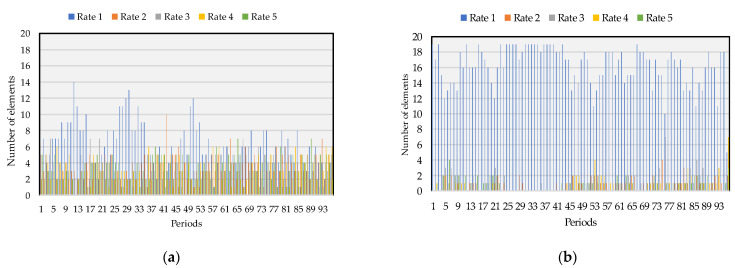
CTR from (**a**) prosumers’ load flexibility per period and (**b**) prosumers’ BES discharging.

Finally, the last remuneration method, considering the rate created by the authors, is the CTR. Table 7 shows the total obtained per rate throughout the day according to the participants’ performance—the actual distribution can be seen in Figure 8. For both cases, rate 1 is the one with more prosumers—mainly on BES discharging, as seen in the blue columns in Figure 8b. Throughout the 96 periods, 584 lower rates were attributed to the load flexibility and 1554 regarding BESS discharging.

The remuneration obtained with this approach can be seen in Table 8. Focusing on the actual flexibility from the participants’ load reduction, as already shown previously, the results are around 160 m.u. for the three case studies. However, when comparing with the results from Table 5, the overall remuneration decreased near 20.27 m.u. Although the ones with better performance receive better compensation, mainly during peak hours, the number of participants with a rate of 5 is still low.

To validate the proposed methodology and support the claim that resources from DR participation in market transactions can bring economic benefits to both players and aggregators, an individual perspective comparing two prosumers with similar behaviors will be analyzed. Although remuneration approach 1 provided low values of total remuneration, which is better from the aggregator perspective, the CTR fair remuneration had a close result to the single fair remuneration and has the advantage of increasing the remuneration by increasing the rate. In the end, this is advantageous for both sides—increasing trustworthiness and giving the proper knowledge regarding community members. With this, the chosen program for this comparison was remuneration approach 4, case study 3.

**Figure 9:**
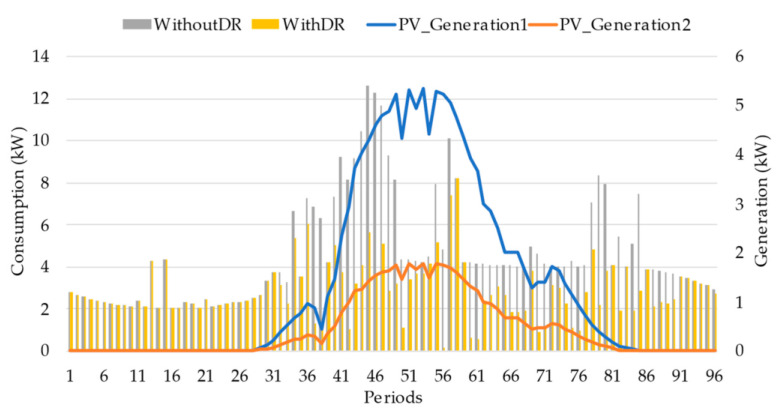
Prosumer ID2 consumption and generation profiles.

**Figure 10:**
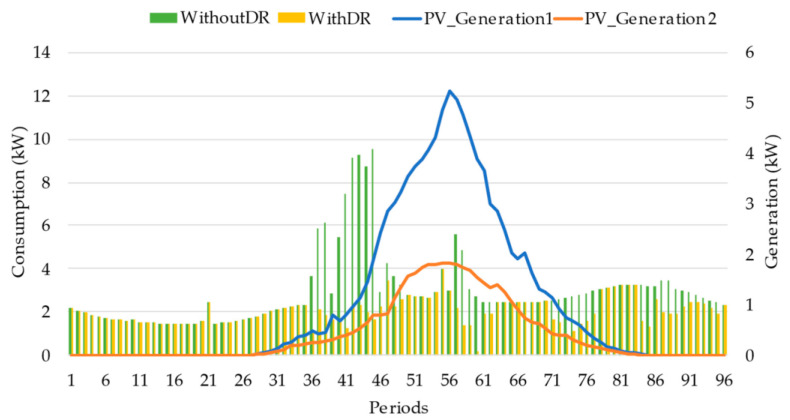
Prosumer ID6 consumption and generation profiles.

Figure 9 and Figure 10 show the comparison-resulting load diagram and generation for prosumer ID2 and prosumer ID6, respectively. It can be seen that the load peak for both prosumers is in a different period from the generation. Participating in the DR event can move their consumption where self-consumption can be applied through their PV generation and saving money, mainly prosumer ID6 from period 36 to 46. Furthermore, prosumer ID 2, from participating in DR events, received a total of 7.58 m.u., and prosumer ID 6 received 8.69 m.u.

## 5. Conclusions

The consumer’s role is changing in the power and energy market. With the smart grid concept being implemented in the real market, their market influence will increase, not only by providing load flexibility, but also by upgrading their portfolio with distributed generation resources, such as renewable bases like photovoltaic panels and battery energy storage systems. For the present paper’s case studies, the authors aim to better understand the influence of the power and energy price on community resource scheduling and their willingness to move their load consumption by participating in demand response events.

From the results obtained in the study presented in this paper, the prosumers’ flexibility to participate was not impacted by the energy price fluctuation for different schedules. The residential prosumers and their reluctance to change their behavior to avoid jeopardizing their comfort are still a complex problem. Although flexibility was provided, it was expected to have a higher level of demand response participation, since the energy prices increased for several periods. Nevertheless, the authors know that higher participation levels will move load consumption to other periods. This fact must be well managed to avoid a rebound effect after the demand response event, considering that prosumers will also reduce the load requested to the grid and, sometimes, help to balance the generation resources associated by selling their excess, namely from battery energy storage. Regarding battery energy storage discharging flexibility, the response to energy price changes in the scheduling phase was observed for this case. Although photovoltaic generation was also considered in these case studies, battery energy storage is much more predictable and controllable, which is useful for load consumption satisfaction for both the owner and the community-associated.

Regarding the perspective of remuneration, although the single approach had the lower values of remuneration, the authors believe that the approach with a rate that classifies the performance from the demand response participants can benefit from the aggregator perspective without jeopardizing the fairness—the ones with a higher level of trustworthiness will still have higher values of compensation. Furthermore, this approach will also motivate continuous participation to increase the rate assigned and have better benefits, sometimes not paying for the energy prices, since their compensation from the flexibility provided may be higher.

To conclude, the authors summarize the results found in this study and compare them with the current state of the art reviewed previously (Table 1):

- On the simulated scheduling results, the contextual energy price did not much change the behavior from the prosumer load flexibility. However, BES was used several times when PV generation was low, as mentioned [16,17].
- Single fair remuneration was the one with the lowest total value of remuneration. From the community manager’s perspective, fair remuneration can still be applied, since the participants will receive higher compensation values for DR participation. In [20], single fair remuneration was also considered in the case studies, but not for prosumers.
- Clustering fair remuneration was the one with the higher total value of remuneration. From the prosumer perspective, aggregating the participants into three groups might be interesting, as providing higher flexibility can help them join different groups. This approach was also considered in [21] but might be highly costly for the community manager.
- CTR fair remuneration had results close to the single fair remuneration plus and has the advantage of increasing the remuneration by increasing the rate. This is advantageous for both sides, increasing trustworthiness and giving the proper knowledge regarding community, as in [19].

In future works, the authors will add electric vehicles as prosumer resources for other types of consumers, such as office buildings. Furthermore, one factor that might impact the results and was not considered was the ramp period and the number of cycles of charge and discharge, which can be considered a disadvantage of the proposed methodology.

## Figures and Tables

**Table 1:** The literature review summarized (✓ means that the topic is covered by the reference).

Ref.	Prosumers	Demand Response	Energy Storage System	Horizon	Remuneration
[12]	-	-	✓	Real-time	-
[13]	-	-	✓	Real-time	-
[15]	-	-	✓	Real-time	-
[17]	✓	-	✓	-	-
[18]	-	Incentive	✓	Real-time	-
[19]	-	Incentive	-	Planning	✓
[20]	-	Incentive	-	Planning	✓
[21]	-	Incentive	-	Planning	-
This work	✓	Incentive and price	✓	Planning and Real-time	✓

**Table 2:** Energy tariffs for the scheduling phase.

Tariff	Peak (m.u./kWh)	Off-Valley (m.u./kWh)	Valley (m.u./kWh)
Flat	0.1815	0.1815	0.1815
Bi-hourly	0.1865	0.1865	0.1669
Tri-hourly	0.2724	0.1686	0.1561

**Table 3:** Consumer contextual trustworthy rate tariffs applied.

Rate	Peak (m.u./kWh)	Off-Valley (m.u./kWh)	Valley (m.u./kWh)
1	0.1815	0.1686	0.1561
2	0.2042	0.1718	0.1625
3	0.2270	0.1751	0.1688
4	0.2497	0.1783	0.1752
5	0.2724	0.1815	0.1815

**Table 4:** Total remuneration according to a single approach.

Case Study	Actual Flexibility (m.u.)	BESS Discharging (m.u.)
1	156.84	4.45
2	157.76	4.41
3	163.81	4.53

**Table 5:** Total remuneration according to clustering method.

Case Study	Actual Flexibility (m.u.)			Total	BES Discharging (m.u.)			Total
	Group 0	Group 1	Group 2		Group 0	Group 1	Group 2	
1	33.22	134.67	28.38	196.27	2.75	2.21	0.84	5.80
2	33.22	134.67	28.38	196.27	2.75	0.87	2.20	5.82
3	33.22	134.67	28.38	196.27	1.08	2.40	2.22	5.70

**Table 6:** Performance results from the classification methods.

Classification Methods	Actual Flexibility		BES Discharging	
	Accuracy	MAE	Accuracy	MAE
Decision Tree	0.10588	0.42105	0.41651	0.50877
K-nearest neighbors	0.83333	0.33333	0.41666	0.50877
Artificial Neural Networks	0.57780	-	0.37780	-

**Table 7:** The number of participants per rate throughout the day.

	Actual Flexibility (m.u.)					BES Discharging (m.u.)				
Rate	1	2	3	4	5	1	2	3	4	5
Participants	584	334	302	290	314	1554	65	83	60	62

**Table 8:** Total remuneration according to the CTR method.

Case Study	Actual Flexibility (m.u.)					Total	BES Discharging (m.u.)					Total
	1	2	3	4	5		1	2	3	4	5	
1	28.56	34.67	30.01	30.80	36.14	160.16	0.85	0.79	1.09	0.84	0.88	4.45
2	28.56	34.67	30.01	30.80	36.14	160.16	0.91	0.77	1.09	0.84	0.87	4.49
3	28.56	34.67	30.01	30.80	36.14	159.63	0.91	0.77	1.09	0.84	0.87	4.49

## Data Availability

https://zenodo.org/record/7277686#.Y3Tj2uzP23I (accessed on 3 November 2022).

## Nomenclature

$C_{\left(t\right)}^{grid_{in}}$	Cost of selling on period *t* (m.u./kW)
$C_{\left(t\right)}^{grid_{out}}$	Cost of buying on period *t* (m.u./kW)
$E_{\left(s,t\right)}^{stor}$	State of charge from energy storage *s* on period *t* (kW)
$E_{\left(s,t\right)}^{stormax}$	Maximum state of charge from energy storage *s* on period *t* (kW)
$E_{\left(s,t\right)}^{stormin}$	Minimum state of charge from energy storage *s* on period *t* (kW)
$P_{\left(c,t\right)}^{DR}$	Flexibility provided by prosumer *c* on period *t* (kW)
$P_{\left(c,t\right)}^{DRmax}$	Maximum flexibility provided by prosumer *c* on period *t* (kW)
$P_{\left(t\right)}^{grid_{in}}$	Power sold from External Supplier on period *t* (m.u./kW)
$P_{\left(t\right)}^{grid_{out}}$	Power bought to External Supplier on period *t* (m.u./kW)
$P_{\left(t\right)}^{gridmax_{in}}$	Maximum power that can be bought from External Supplier on period *t* (m.u./kW)
$P_{\left(t\right)}^{gridmax_{out}}$	Maximum power that can be bought from External Supplier on period *t* (m.u./kW)
$P_{\left(c,t\right)}^{DR}$	Power from Demand Response active consumer *c* on period *t* (kW)
$P_{\left(p,t\right)}^{PV}$	Power from Distributed Generation p on period *t* (kW)
$P_{\left(s,t\right)}^{ch}$	Power from charging Energy Storage System b on period *t* (kW)
$P_{\left(s,t\right)}^{dch}$	Power from discharging Energy Storage System b on period *t* (kW)
$P_{\left(t\right)}^{grid}$	Power from External Supplier on period *t* (kW)
$P_{\left(t\right)}^{load}$	Initial Load on period *t* (kW)
$W_{\left(c,t\right)}^{DR}$	Flexibility weight from prosumer *c* on period t
$X_{\left(c,t\right)}^{DR}$	Availability from prosumer *c* on period *t*
$X_{\left(s,t\right)}^{ch}$	Charging status from Energy Storage System *b* on period t
$X_{\left(s,t\right)}^{dch}$	Discharging status from Energy Storage System *b* on period t

## Appendix A

The dataset used in the present paper was published on Zenodo (https://zenodo.org/record/7277686, accessed on 3 November 2022).

## References

[1] Ramos D., Faria P., Vale Z., Correia R. (2022). Short Time Electricity Consumption Forecast in an Industry Facility. IEEE Trans. Ind. Appl..

[2] Fei L., Shahzad M., Abbas F., Muqeet H.A., Hussain M.M., Bin L. (2022). Optimal Energy Management System of IoT-Enabled Large Building Considering Electric Vehicle Scheduling, Distributed Resources, and Demand Response Schemes. Sensors.

[3] Bhattacharya S., Chengoden R., Srivastava G., Alazab M., Javed A.R., Victor N., Maddikunta P.K.R., Gadekallu T.R. (2022). Incentive Mechanisms for Smart Grid: State of the Art, Challenges, Open Issues, Future Directions. Big Data Cogn. Comput..

[4] Cheng L., Yin L., Wang J., Shen T., Chen Y., Liu G., Yu T. (2021). Behavioral decision-making in power demand-side response management: A multi-population evolutionary game dynamics perspective. Int. J. Electr. Power Energy Syst..

[5] Muqeet H.A., Javed H., Akhter M.N., Shahzad M., Munir H.M., Nadeem M.U., Bukhari S.S.H., Huba M. (2022). Sustainable Solutions for Advanced Energy Management System of Campus Microgrids: Model Opportunities and Future Challenges. Sensors.

[6] Halbe S., Chowdhury B., Abbas A. Mitigating Rebound Effect of Demand Response using Battery Energy Storage and Electric Water Heaters. Proceedings of the 2019 IEEE 16th International Conference on Smart Cities: Improving Quality of Life Using ICT & IoT and AI (HONET-ICT).

[7] Barik A.K., Das D.C. (2021). Integrated resource planning in sustainable energy-based distributed microgrids. Sustain. Energy Technol. Assessments.

[8] Estebsari A., Mazzarino P.R., Bottaccioli L., Patti E. (2022). IoT-Enabled Real-Time Management of Smart Grids With Demand Response Aggregators. IEEE Trans. Ind. Appl..

[9] Zhu N., Gao C., Lu T., Liu F., Han Y., Zhang J. Assistant analyzer for the characteristics of electricity behavior based on big data technology. Proceedings of the 5th IEEE International Conference on Electric Utility Deregulation, Restructuring and Power Technologies, DRPT 2015.

[10] Oprea S.-V., Bâra A., Tudorică B.G., Călinoiu M.I., Botezatu M.A. (2021). Insights into demand-side management with big data analytics in electricity consumers’ behaviour. Comput. Electr. Eng..

[11] Pham Q.-V., Liyanage M., Deepa N., VVSS M., Reddy S., Maddikunta P.K.R., Khare N., Gadekallu T.R., Hwang W.-J. (2021). Deep Learning for Intelligent Demand Response and Smart Grids: A Comprehensive Survey. arXiv.

[12] Khojasteh M., Faria P., Vale Z. (2022). Scheduling of battery energy storages in the joint energy and reserve markets based on the static frequency of power system. J. Energy Storage.

[13] Khojasteh M., Faria P., Vale Z. (2022). A robust model for aggregated bidding of energy storages and wind resources in the joint energy and reserve markets. Energy.

[14] Yao J., Venkitasubramaniam P. Stochastic games of end-user energy storage sharing. Proceedings of the 2016 IEEE 55th Conference on Decision and Control (CDC).

[15] Pandey A.K., Jadoun V.K., Sabhahit J.N. (2022). Real-Time Peak Valley Pricing Based Multi-Objective Optimal Scheduling of a Virtual Power Plant Considering Renewable Resources. Energies.

[16] Sijakovic N., Terzic A., Fotis G., Mentis I., Zafeiropoulou M., Maris T.I., Zoulias E., Elias C., Ristic V., Vita V. (2022). Active System Management Approach for Flexibility Services to the Greek Transmission and Distribution System. Energies.

[17] Calearo L., Ziras C., Sevdari K., Marinelli M. Comparison of Smart Charging and Battery Energy Storage System for a PV Prosumer with an EV. Proceedings of the 2021 IEEE PES Innovative Smart Grid Technologies Europe: Smart Grids: Toward a Carbon-Free Future, ISGT Europe 2021.

[18] Arasteh F., Riahy G.H. (2019). MPC-based approach for online demand side and storage system management in market based wind integrated power systems. Int. J. Electr. Power Energy Syst..

[19] Silva C., Faria P., Vale Z., Terras J.M., Albuquerque S. (2022). Rating the participation in Demand Response events with a contextual approach to improve accuracy of aggregated schedule. Energy Rep..

[20] Silva C., Faria P., Vale Z. Aggregation of Prosumers in the Context of Demand Response and Distributed Generation Remuneration and Scheduling. Proceedings of the 2020 IEEE/PES Transmission and Distribution Conference and Exposition (T&D).

[21] Faria P., Spinola J., Vale Z. (2016). Aggregation and Remuneration of Electricity Consumers and Producers for the Definition of Demand-Response Programs. IEEE Trans. Ind. Inform..

[22] Silva C., Faria P., Vale Z., Corchado J. (2022). Demand response performance and uncertainty: A systematic literature review. Energy Strat. Rev..

[23] Silva C., Faria P., Vale Z. (2019). Multi-Period Observation Clustering for Tariff Definition in a Weekly Basis Remuneration of Demand Response. Energies.

[24] Silva C., Faria P., Vale Z., García Bringas P., Pérez García H., Martinez-de-Pison F.J., Villar Flecha J.R., Troncoso Lora A., de la Cal E.A., Herrero Á., Martínez Álvarez F., Psaila G., Quintián H. (2023). DR Participants’ Actual Response Prediction Using Artificial Neural Networks. Proceedings of the 17th International Conference on Soft Computing Models in Industrial and Environmental Applications (SOCO 2022).

[25] Ribeiro B., Pereira H., Gomes L., Vale Z., Bringas P.G., Garc’ia H.P., de Pisón F.J.M., Flecha J.R.V., Lora A.T., de la Cal E.A., Herrero Á., Mart’inez-Álvarez F., Psaila G., Quintián H. (2022). Python-Based Ecosystem for Agent Communities Simulation. Proceedings of the 17th International Conference on Soft Computing Models in Industrial and Environmental Applications ({SOCO} 2022).

[26] Yang Y., Bremner S., Menictas C., Kay M. (2022). Modelling and optimal energy management for battery energy storage systems in renewable energy systems: A review. Renew. Sustain. Energy Rev..

